# Cloning and Heterologous Expression of a Large-sized Natural Product Biosynthetic Gene Cluster in *Streptomyces* Species

**DOI:** 10.3389/fmicb.2017.00394

**Published:** 2017-03-15

**Authors:** Hee-Ju Nah, Hye-Rim Pyeon, Seung-Hoon Kang, Si-Sun Choi, Eung-Soo Kim

**Affiliations:** Department of Biological Engineering, Inha UniversityIncheon, South Korea

**Keywords:** *Streptomyces*, natural product, biosynthetic gene cluster, heterologous expression, large-sized

## Abstract

Actinomycetes family including *Streptomyces* species have been a major source for the discovery of novel natural products (NPs) in the last several decades thanks to their structural novelty, diversity and complexity. Moreover, recent genome mining approach has provided an attractive tool to screen potentially valuable NP biosynthetic gene clusters (BGCs) present in the actinomycetes genomes. Since many of these NP BGCs are silent or cryptic in the original actinomycetes, various techniques have been employed to activate these NP BGCs. Heterologous expression of BGCs has become a useful strategy to produce, reactivate, improve, and modify the pathways of NPs present at minute quantities in the original actinomycetes isolates. However, cloning and efficient overexpression of an entire NP BGC, often as large as over 100 kb, remain challenging due to the ineffectiveness of current genetic systems in manipulating large NP BGCs. This mini review describes examples of actinomycetes NP production through BGC heterologous expression systems as well as recent strategies specialized for the large-sized NP BGCs in *Streptomyces* heterologous hosts.

## Introduction

Natural products (NPs) and their derivatives lead a huge pharmaceutical market share comprising 61% of anticancer drugs and 49% of anti-infection medicine in the past 30 years (Newman and Cragg, 2012). Especially, actinomycetes NPs are a major resource for drug discovery and development, mainly due to their structural novelty, diversity, and complexity (Donadio et al., 2007). Isolation and characterization of NP biosynthetic gene clusters (BGCs) have further accelerated our understanding of their molecular biosynthetic mechanisms, leading to the rational redesign of novel NPs through BGC manipulation (Fischer et al., 2003; Castro et al., 2015).

Some of these potentially valuable BGCs are, however, derived from non-culturable meta-genomes or genetically recalcitrant microorganisms. Moreover, many of these BGCs are expressed poorly or not at all under laboratory culture conditions, which makes it challenging to characterize the target NPs (Galm and Shen, 2006). Since efficient expression of actinomycetes NP BGCs present a major bottleneck for novel NP discovery, various cryptic BGC awakening strategies such as regulatory genes control, ribosome engineering, co-culture fermentation, and heterologous expression have been pursued for NP development (Tang et al., 2000; Flinspach et al., 2014; Martinez-Burgo et al., 2014; Miyamoto et al., 2014).

A traditional method for BGC cloning involves cosmid library construction by partial digestion or random shearing of chromosomal DNA. A typical size of NP BGC is usually larger than 20 kb (sometimes over 100 kb), and a cosmid vector system can only accept a relatively small BGC (up to 40 kb) or only a part of a large BGC. Therefore, cloning and efficient overexpression of an entire BGC still remains challenging due to the ineffectiveness of current host cells including the genetic and metabolic characteristics in manipulating large BGCs for heterologous expression. This mini review summarizes the list of the actinomycetes NP BGCs that have been successfully cloned and expressed in *Streptomyces* heterologous hosts (Table 1). In addition, three cloning and heterologous expression systems, which are quite suitable for large NP BGCs, such as transformation-associated recombination (TAR) system, integrase-mediated recombination (IR) system, and plasmid *Streptomyces* bacterial artificial chromosome (pSBAC) system are introduced (Figure 1).

**Table 1:** **Heterologous expression of NP BGCs**.

**NP name (Class)**	**Original host**	**BGC size (kb)**	**Expression method**	**Heterologous host**	**WT titer (mg/L)**	**HH titter (mg/L)**	**References**
A201A (Nucleoside)	*Sacchaothrix mutabilis* subsp. Capreolus	34	PAC Integrative	*S. coelicolor S. lividans*	12	8	Saugar et al., 2016
A54145 (NRPS)	*S. fradiae* NRRL 18160	~60	BAC Integrative	*S. ambofaciens S. roseosporus*	NR	100 ~ 385	Alexander et al., 2010
Actinorhodin (PKS II)	*S. coelicolor* M145	33	LLHR Integrative	*Streptomyces*	NR	NR	Chen and Qin, 2011
Amicetin (NRPS)	*S. vinaceusdrappus* NRRL 2363	37.3	Cosmid Replicative	*S. lividans*	NR	NR	Zhang et al., 2012
Ammosamides A-C (Alkaloid)	*S*. sp. CNR-698	35	TAR Integrative	*S. coelicolor*	4 ~ 6	17	Jordan and Moore, 2016
Anthracimycin (PKS I)	*S*. sp. T676	53.2	PAC Integrative	*S. coelicolor*	NR	8.6 ~ 13.8	Alt and Wilkinson, 2015
Aristeromycin (Nucleoside)	*S. citricolor*	37.5	Cosmid Replicative	*S. albus*	NR	ND	Kudo et al., 2016
Aureothin (PKS I)	*S. thioluteus* HKI-227	27	Cosmid Integrative	*S. lividans*	NR	NR	He and Hertweck, 2003
Barbamide (PKS-NRPS)	*Moorea producens*	26	LCHR Replicative	*S. venezuelae*	NR	ND^*^	Kim et al., 2012
Bernimamycin (Thiopeptide)	*S. bernensis* UC5144	12.9	LLHR Integrative	*S. lividans S. venezuelae*	NR	NR	Malcolmson et al., 2013
Blasticidin S (Nucleoside)	*S. griseochromogenes*	20	Cosmid Replicative	*S. lividans*	NR	NR	Cone et al., 2003
Cacibiocin (Aminocoumarin)	*Catenulispora acidiphila*	20	LLHR Integrative	*S. coelicolor*	4.9	60	Zettler et al., 2014
Caerulomycin (PKS-NRPS)	*Actinoalloteichus cyanogriseus* WH1-2216-6	44.6	Cosmid Replicative	*S. coelicolor*	NR	NR	Zhu et al., 2012
Cephamycin C (NRPS)	*S. clavuligerus* ATCC 27064	35.6	Cosmid Integrative	*S. flavogriseus S. coelicoor S. albus*	3640	8 ~ 300^#^	Martinez-Burgo et al., 2014
Chalcomycin (PKS I)	*S. bikiniensis*	80	LLHR Integrative	*S. fradiae*	NR	NR	Ward et al., 2004
Chaxamycin (PKS I)	*S. leeuwenhoekii*	80.2	PAC Integrative	*S. coelicolor*	NR	NR	Castro et al., 2015
Chloramphenicol (PKS-NRPS)	*S. venezuelae* ATCC10712	NR	Cosmid Integrative	*S. coelicolor*	NR	1.6 ~ 26.23	Gomez-Escribano and Bibb, 2011
Chlorizidine A (PKS I)	*S*. sp. CNH-287	42.4	Fosmid Integrative	*S. coelicolor*	NR	NR	Mantovani and Moore, 2013
Chrysomycin (PKS II)	*S. albaduncus* AD0819	34.65	Cosmid Replicative	*S. lividans*	NR	ND	Kharel et al., 2010
Clavulanic acid (β-lactam)	*S. clavuligerus* ATCC27064	20	Cosmid Integrative	*S. flavogriseus S. coelicolor*	164.50	0.6	Alvarez-Alvarez et al., 2013
Complestatin (Glycopeptide)	*S. chartreusis* AN1542	54.5	LLHR Integrative	*S. lividans*	5.57	0.24	Park et al., 2016
Congocidine (NRPS)	*S. ambofaciens* ATCC23877	NR	Cosmid Integrative	*S. coelicolor*	NR	NR	Gomez-Escribano and Bibb, 2011
Coumermycin A1 (Aminocoumarin)	*S. rishiriensis* DSM40489	38.6	Cosmid Integrative	*S. coelicolor*	0.002 ~ 0.005	0.01	Wolpert et al., 2008
Cremeonycin (Diazoquinone)	*S. cremeus* NRRL3241	18	BAC Integrative	*S. lividans*	NR	NR	Waldman et al., 2015
Cyclothiazomycin (Thiopeptide)	*S. hygroscopicus* 10-22	22.7	LLHR Integrative	*S. lividans*	NR	NR	Wang et al., 2010
Daptomycin (NRPS)	*S. roseosporus* NRRL 11379	128	BAC Integrative	*S. lividans*	900	18	Miao et al., 2005
Desotamide (NRPS)	*S. scopuliridis* SCSIO	39	Cosmid Integrative	*S. coelicolor*	NR	ND^*^	Li et al., 2015
Epothilone (PKS-NRPS)	*Sorangium cellulosum* SHP44	56	LLHR Replicative & Integrative	*S. coelicolor*	0.05 ~ 0.1	20	Tang et al., 2000
FK506 (PKS I)	*S*. sp. KCCM11116P	120	LCHR Integrative	*S. albus*	NR	NR	Chen et al., 2014
	*S. tsukubaensis*	83.5	PAC Integrative	*S. coelicolor*	1.20	5.50	Jones et al., 2013
Flustatin (PKS II)	*Micromonospora* SCSIO N160	40	Cosmid Replicative	*S. coelicolor*	NR	NR	Yang et al., 2015
Fostriecin PKS (PKS I)	*S. pulveraceus* ATCC31906	48.6	LLHR Replicative & Integrative	*S. coelicolor S. lividans*	NR	ND	Su et al., 2015
Galbonolide B (PKS I)	*S*. sp. L235	12.1	LLHR Integrative	*S. coelicolor*	NR	NR^†^	Liu et al., 2015
GE2270 (Thiopeptide)	*Planobispora rosea* ATCC53733	21.4	LLHR Integrative	*S. coelicolor*	NR	0.08	Flinspach et al., 2014
GE37468 (Thiazolyl peptide)	*S*. ATCC 55365	17.1	LLHR Integrative	*S. lividans*	5 ~ 7	2 ~ 3	Young and Walsh, 2011
Gilvocarcin V (PKS II)	*S. griseoflavus* Gö 3592	32.9	Cosmid Replicative	*S. lividans*	20 ~ 30	NR	Fischer et al., 2003
Goadsporin (Azole)	*S*. sp. TP-A0584	14	LLHR Integrative	*S. lividans*	126.3	342.7	Haginaka et al., 2014
Gougerotin (Nucleoside)	*S. graminearus*	28.7	LCHR Integrative	*S. coelicolor*	NR	NR	Niu et al., 2013
Granaticin (PKS II)	*S. violaceoruber* Tü22	39	Cosmid Replicative	*S. coelicolor*	NR	NR	Ichinose et al., 1998
Grecocycline (PKS II)	*S*. sp. Acta 1362	36	TAR Integrative	*S. albus*	NR	ND^*^	Bilyk et al., 2016
Grincamycin (PKS II)	*S. lusitanus* SCSIO LR32	37	LCHR Integrative	*S. coelicolor*	NR	ND^*^	Zhang et al., 2013
Holomycin (NRPS)	*S. clavuligerus* ATCC27064	24	LLHR Integrative	*S. coelicolor*	NR	NR	Robles-Reglero et al., 2013
Kanamycin (Aminoglycoside)	*S. kanamyceticus* ATCC12853	32	Cosmid Replicative	*S. venezuelae*	1.80	0.50	Thapa et al., 2007
Kinamycin (PKS II)	*S. murayamaensis*	40	Cosmid Replicative	*S. lividans*	NR	ND	Gould et al., 1998
Lincomycin (Lincosamide)	*S. lincolnensis* ATCC25466	35	Cosmid Integrative	*S. coelicolor*	50.1	0.66 ~ 1.49	Koberska et al., 2008
Lyngbyatoxin A (NRPS)	*Moorea products*	11.3	LLHR Replicative	*S. coelicolor*	NR	NR	Jones et al., 2012
Lysolipin (PKS II)	*S. tendae* Tü 4042	43.2	Cosmid Replicative	*S. albus*	NR	NR	Lopez et al., 2010
Macrotetrolide (PKS II)	*S. griseus* DSM40695	25	LLHR Integrative	*S. lividans*	40	10	Kwon et al., 2001
Marineosin (Oligopyrrole)	*S*. sp. CNQ-617	32	Cosmid Integrative	*S. venezuelae*	0.5	5	Salem et al., 2014
Medermycin (PKS II)	*S*. sp. AM7161	30	LLHR Integrative	*S. coelicolor S. lividans*	NR	NR	Ichinose et al., 2003
	*S*. sp. K73	36.2	Cosmid Replicative	*S. coelicolor*	NR	NR	Ichinose et al., 2003
Mensacarcin (PKS II)	*S. bottropensis*	40	Cosmid Integrative	*S. albus*	NR	ND^*^	Yan et al., 2012
Meridamycin (PKS-NRPS)	*S*. sp. NRRL 30748	90	pSBAC Integrative	*S. lividans*	NR	0.1^#^	Liu et al., 2009
Merochlorin A-D (PKS-terpenoid)	*S*. sp. CNH-189	57.6	Fosmid Integrative	*S. coelicolor*	10.0	NR	Kaysser et al., 2012
Mycosperine	*Actinosynnema mirum* DSM43827	6.3	LLHR Integrative	*S. avermitilis*	NR	NR^#^	Miyamoto et al., 2014
Naphthocyclinone (PKS II)	*S. arenae* DSM40737	12	Cosmid Replicative	*S. coelicolor*	NR	NR	Brunker et al., 1999
Nataxazole (PKS I)	*S*. sp. Tü6176	44.1	TAR Integrative	*S. lividans*	NR	ND^*^	Cano-Prieto et al., 2015
Neocarzilin (PKS I)	*S. carzinostaticus* var. F-41	33	Cosmid Integrative	*S. lividans*	NR	NR	Otsuka et al., 2004
Nogalamycin (PKS II)	*S. nogalater*	20	Cosmid Replicative	*S. lividans*	NR	NR	Ylihonko et al., 1996
		29	LLHR Replicative	*S. lividans S. galilaeus S. peucetius*	NR	NR	Torkkell et al., 2001
Novobiocin (Aminocoumarin)	*S. spheroides*	25.6	Cosmid Replicative	*S. lividans*	NR	NR^†^	Steffensky et al., 2000
Oleandomycin (PKS I)	*S. antibiticus*	65	LLHR Replicative	*S. lividans*	NR	NR^†^	Shah et al., 2000
Oxytetracycline (PKS II)	*S. rimosus* M4018	29	Cosmid Integrative	*S. venezuelae*	75	431	Yin et al., 2016
	*S. rimosus*	34	Cosmid Replicative	*S. lividans*	NR	20	Binnie et al., 1989
Phosphinothricin (NRPS)	*S. viridochromogenes* DSM 40736	40	Fosmid Integrative	*S. lividans*	NR	NR	Blodgett et al., 2005
Puromycin (Nucleoside)	*S. alboniger*	13	Cosmid Replicative	*S. lividans S. griseofuscus*	150.00	4 ~ 15	Lacalle et al., 1992
R1128 (PKS II)	*S*. sp. R1128	17	Cosmid Replicative	*S. lividans*	NR	1.00	Marti et al., 2000
Ravidomycin PKS II	*S. ravidus*	33.28	Cosmid Replicative	*S. lividans*	NR	NR	Kharel et al., 2010
Rebeccamycin (Indolocarbazole)	*Saccharothrix aerocolonigenes* ATCC 39243	25.6	Cosmid Replicative	*S. albus*	NR	NR	Sanchez et al., 2002
Resorcinomycin	*Streptorerticilium roseoverticillatum*	11	LLHR Replicative	*S. lividans*	NR	ND^*^	Ooya et al., 2015
Rimosamide (NRPS-PKS)	*S. rimosus* NRRL B-2659	30.5	Fosmid Integrative	*S. lividans*	NR	NR	McClure et al., 2016
Rishirilide A (PKS II)	*S. bottropensis*	50	Cosmid Integrative	*S. albus, S. lividans*	NR	NR	Yan et al., 2012
Salinomycin (PKS I)	*S. albus* DSM41398	106	LLHR Integrative	*S. coelicolor*	NR	NR	Yin et al., 2015
Sparsomycin (NRPS)	*S. sparsogenes*	30	TAR Integrative	*S. lividans*	NR	NR	Rui et al., 2015
Staurosporine (Indolocarbazole)	*S. sanyensis* FMA	34.6	Cosmid Integrative	*S. coelicolor*	NR	NR	Li T. et al., 2013
	*S*. sp. TP-A0274	20	Cosmid Integrative	*S. lividans*	10.5	2.6	Onaka et al., 2002
Streptocolin (Lanthipeptide)	*S. colimus* Tü365	6	Cosmid Integrative	*S. coelicolor*	NR	5.4 ~ 110	Iftime et al., 2015
Streptothricin (NRPS)	*S. sp*. TP-A0356	41	Cosmid Replicative	*S. coelicolor*	NR	NR	Li J. et al., 2013
Tautomycetin (PKS I)	*S*. sp. CK4412	80	pSBAC Integrative	*S. coelicolor S. lividans*	3.10	3.91 ~ 4.05	Nah et al., 2015
Tetracenomycin C (PKS II)	*S. glaucescens*	24	LLHR Replicative	*S. lividans*	NR	NR	Motamedi and Hutchinson, 1987
Tetrangulol (PKS II)	*S*. sp. WP4669 *S. rimosus N*RRL3016	40	Cosmid Replicative	*S. lividans*	NR	NR	Hong et al., 1997
Thioriridamide (Ribosomal peptide)	*S. olivoriridis* NA05001	14.5	LLHR Replicative	*S. lividans*	NR	NR	Izawa et al., 2013
		70	BAC Integrative	*S. avermitilis*	NR	2.5	Izumikawa et al., 2015
TP-1161 (Thiopeptide)	*Nocardiopsis* sp. TFS65-07	16	Cosmid Replicative	*S. coelicolor*	NR	ND	Engelhardt et al., 2010
Undecylprodigiosin (NRPS)	*S. coelicolor* M145	38	LLHR Replicative	*S. parvulus*	NR	NR	Malpartida et al., 1990
Validamycin (Pseudosaccharide)	*S. hygroscopicus* var. limoneus KTCC 1715	37	Cosmid Replicative	*S. lividans S. albus*	NR	NR	Singh et al., 2006
Venemycin (PKS I)	*S. venezuelae*	29.5	Cosmid Integrative	*S. coelicolor*	NR	ND	Thanapipatsiri et al., 2016
Versipelostatin (PKS I)	*S. versipellis* 4083	108	BAC Integrative	*S. albus*	1.5	21.0	Hashimoto et al., 2015
YM-216391 (NRPS)	*S. nobilis*	<40	Cosmid Replicative	*S. lividans*	NR	0.18	Jian et al., 2012

PKS, polyketide synthase; NRPS, non-ribosomal peptide synthase; S, Streptomyces; sp, species; TAR, transformation-associated recombination; PAC, phage P1 artificial chromosome; BAC, bacterial artificial chromosome; LLHR, linear-plus-linear homologous recombination; LCHR, linear-plus-circular homologous recombination; NR, not reported (but produced); ND, not detected (not produced); WT, wild type; HH, heterologous host;

* intermediate produced only;

† expressed part of gene cluster;

# *produced by gene cluster modification (e.g., Promoter substitution)*.

**Figure 1:**
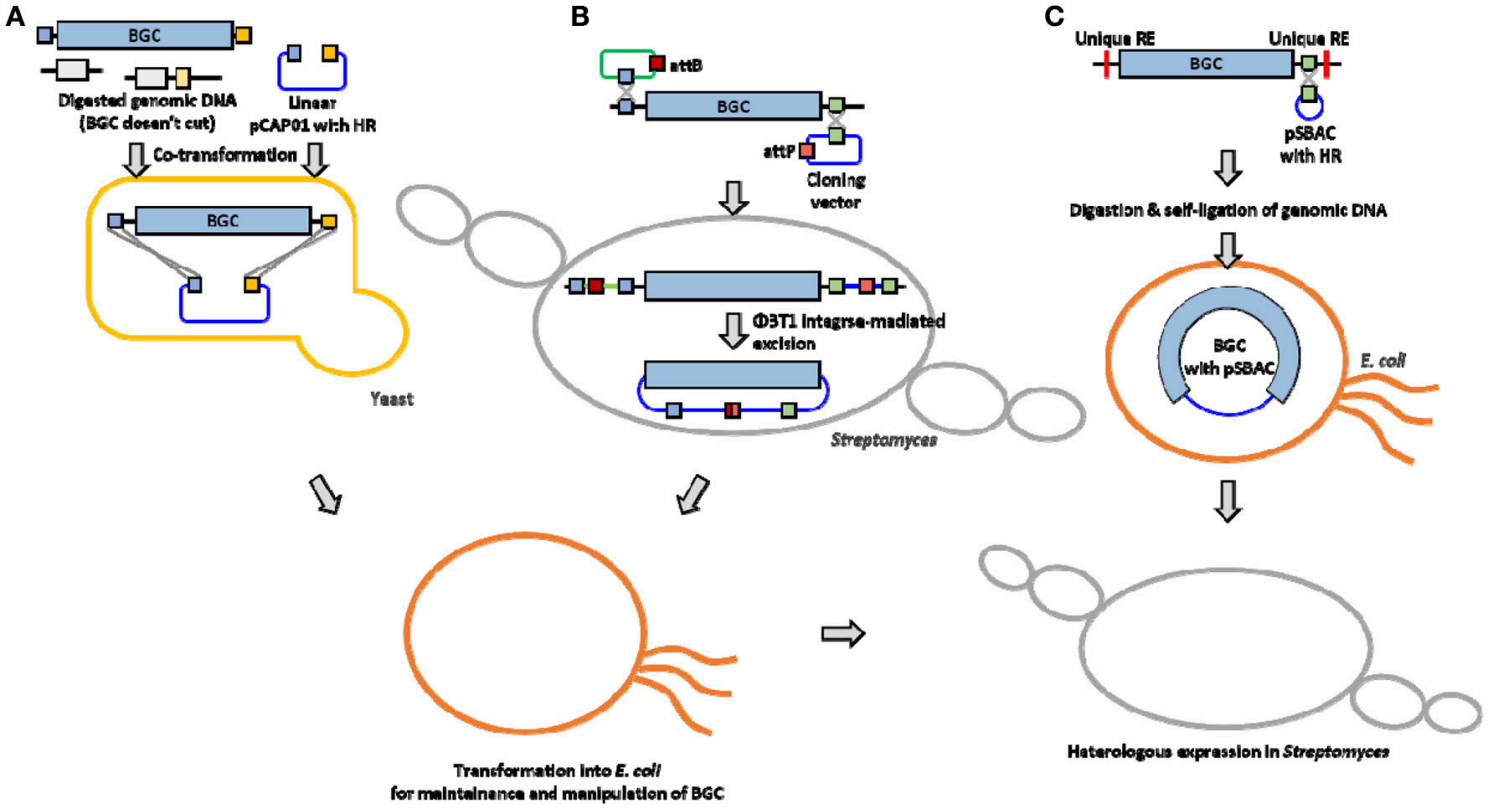
**Overview of large BGC cloning system (A)** TAR system **(B)** IR system **(C)** pSBAC system. HR, Homologous region; RE, restriction enzyme.

## Traditional method for heterologous expression of NP BGCs

We summarized about 90 actinomycetes NP BGCs that have been successfully expressed in *Streptomyces* heterologous hosts from the last several decades (Table 1). Relatively small BGCs encoding Type II polyketide were first to be isolated at the beginning of heterologous expression research. Many of the listed BGCs (about 83%) were isolated by cosmid/fosmid library construction and some of these BGCs were cloned into replicative or integrative vector by linear-plus-linear (recombination between two linear DNAs) or linear-plus-circular (recombination between linear and replicating circular DNA) homologous recombination. Approximately 60% of BGCs were integrated into the heterologous host chromosome and only 37% of BGCs existed in the heterologous host via replicative plasmid. Cosmid vectors such as pOJ446 and SuperCos1 were used to be replicative or integrative in the heterologous host, so the production level of the heterologously expressed NP BGC varied significantly. Some BGCs were isolated with two different vector systems, followed by heterologous expression via both integrative and replicative systems. For example, the epothilone BGC was expressed by both pSET152-based integration vector and SCP2^*^-based replication vectors, so that its expression level was increased from 0.1 mg/L in the original *Sorangium cellulosum* system to 20 mg/L in the epothilone BGC-expressing *Streptomyces* host (Tang et al., 2000). *S. coelicolor* and *S. lividans* were two major strains for heterologous expression, thanks to their well-characterized genetic and biochemical properties. About 12% BGCs were expressed in another popular heterologous host, *S. albus*, which has fast growth and an efficient genetic system (Zaburannyi et al., 2014). Comparing with the original NP producing strains, approximately 14% of NPs had a higher expression level and 12% lower when they were expressed in the heterologous hosts. When bernimamycin BGC was heterologously expressed both in *S. lividans* and *S. venezuelae*, its production yield was increased 2.4-fold in *S. lividans* with no production in *S. venezuelae* (Malcolmson et al., 2013).

## Cloning systems of large NP BGCs for heterologous expression in *Streptomyces*

### TAR system

The TAR system takes advantage of the natural *in vivo* homologous recombination of *Saccharomyces cerevisiae* (Larionov et al., 1994). It has also been applied to capture and express large biosynthetic gene clusters from environmental DNA samples (Feng et al., 2010; Kim et al., 2010). Yamanaka and colleagues designed TAR cloning vector, pCAP01, which consists of three elements, one from each of yeast, *E. coli*, and actinobacteria (Yamanaka et al., 2014). The target BGC can be directly captured and manipulated in yeast background, and the captured BGC can be shuttled between *E. coli* and actinobacteria species. It also has a pUC *ori* that could stably carry an over 50 kb insert in *E. coli* hosts. The pCAP01 vector contains *oriT* and *attP-int* that can transfer the target BGC by conjugation, and the DNA stability can be maintained by insertion into heterologous host chromosomes. To generate a capturing vector, both flanking homologous arms of the target BGC were PCR-amplified and cloned into the pCAP01. The linearized capturing vector and the restriction enzyme digested genomic DNA were co-transformed into yeast, then the target BGC was captured by yeast recombination activities (Figure 1A). The marinopyrrole BGC (30 kb) and the taromycin A BGC (67 kb) were captured by this TAR system, and functionally expressed in *Streptomyces coelicolor* (Yamanaka et al., 2014).

### IR system

Most cloning systems to clone a large DNA fragment directly from bacterial genome are based on different site-specific recombination systems that consist of a specialized recombinase and its target sites. The IR system is based on ΦBT1 integrase-mediated site-specific recombination and simultaneous *Streptomyces* genome engineering (Du et al., 2015). The actinorhodin BGC, the napsamycin BGC and the daptomycin BGC were successfully isolated by the IR system (Du et al., 2015). pUC119-based suicide vector and pKC1139 carrying mutated *attP* or *attB*, respectively, and an integrative plasmid containing the ΦBT1 integrase gene were used for the system (Figure 1B). The pUC119-based plasmid carrying mutated *attB* and a homologous region to 5′ end of the target BGC was introduced into the chromosome by single crossover. The pKC1139 carrying mutated *attP* and a homologous region to 3′ end of the BGC was transferred and integrated into chromosome by conjugation and single crossover through cultivation at high temperature above 34°C. Expression of ΦBT1 integrase leads to excision of the pKC1139 containing the target BGC. The pKC1139 containing BGC from original producing *Streptomyces* was extracted and transferred into *E. coli* for recovery. The IR system was only expressed in parental strain not heterologous host, but it was presumed to be transferred and maintained by replication in heterologous host (Du et al., 2015).

### pSBAC vector system

In the early 1990s, Bacterial Artificial Chromosomes (BAC) was reported to carry inserts approaching 200 kb in length emerged (Shizuya et al., 1992). Various BAC vectors have been used extensively for construction of DNA libraries to facilitate physical genomic mapping and DNA sequencing efforts (Sosio et al., 2000; Martinez et al., 2004; Fuji et al., 2014; Varshney et al., 2014). Several *E. coli-Streptomyces* shuttle BAC vectors have been developed to carry the large-sized NP BGCs such as pStreptoBAC V and pSBAC (Miao et al., 2005; Liu et al., 2009). The utility of pSBAC was demonstrated through the precise cloning and heterologous expression of the tautomycetin BGC and the pikromycin BGC of the type I PKS biosynthetic pathway, as well as the meridamycin BGC of the PKS-NRPS hybrid biosynthetic pathways (Liu et al., 2009; Nah et al., 2015). Unique restriction enzyme recognition sites naturally existing or artificially inserted into both flanking regions of the entire BGC were used for capturing the BGCs. The pSBAC vector was also inserted within the unique restriction enzyme site by homologous recombination. And then the entire target BGC was captured in a single pSBAC through straightforward single restriction enzyme digestion and self-ligation (Figure 1C). The pSBAC contains two replication origins, *ori2* and *oriV*, for DNA stability in *E. coli*, and *oriT* and ΦC31 *attP-int* for BGC integration into the surrogate host chromosome through intergenic conjugation. The recombinant pSBAC containing the large BGCs of varied length from 40 kb to over 100 kb have been successfully cloned and conjugated from *E. coli* to *S. coelicolor* and *S. lividans* (Liu et al., 2009; Nah et al., 2015), implying that the pSBAC system seems to be the most suitable for large BGC cloning comparing with TAR and IR systems.

Recently, a new cloning method named CATCH (Cas9-Assisted Targeting of Chromosome) based on the *in vitro* application of RNA-guided Cas9 nuclease was developed (Jiang and Zhu, 2016). The Cas9 nuclease cleaves target DNA *in vitro* from intact bacterial chromosomes embedded in agarose plugs, which can be subsequently ligated with cloning vector through Gibson assembly. Jiang and colleagues cloned the 36-kb jadomycin BGC from *S. venezuelae* and the 32-kb chlortetracycline BGC from *S. aureofaciens* by CATCH (Jiang et al., 2015).

## *Streptomyces* heterologous expression of NP BGCs

The *Streptomyces* genus is suitable for heterologous expression of large NP BGCs due to its intrinsic ability to produce various valuable secondary metabolites. Well-studied *Streptomyces* strains such as *S. coelicolor, S. lividans*, and *S. albus* have been mainly used as heterologous expression surrogate hosts (Table 1). The regulatory networks of secondary metabolite production have been well characterized in these strains, and thus several NP high-level producing strains have been constructed (Baltz, 2010; Gomez-Escribano and Bibb, 2011). In addition, some of these *Streptomyces* host genomes have been further engineered to eliminate precursor-competing biosynthetic BGCs, so that the extra precursors such as malonyl-CoA and acetyl-CoA could be funneled into the target polyketide NP biosynthesis (Gomez-Escribano and Bibb, 2011).

As shown in Table 1, most of the heterologously expressed NPs were detected as a final product, but some were detected as an intermediate due to their partial BGC expression. The NP production yield was similar to or slightly lower than that in WT. To increase the production level in heterologous hosts, it was devised to substitute with strong promoters or to increase the copy number of BGCs (Montiel et al., 2015; Nah et al., 2015). In case of pSBAC system, the tautomycetin production yield in the heterologous hosts was similar to that in the original producing strain. The selection marker on the tautomycetin BGC was changed and re-introduced into the heterologous host by tandem repeat, resulting in further yield increase from 3.05 to 13.31 mg/L in comparison with the heterologous host harboring only single copy of tautomycetin BGC. The heterologous host harboring tandem copies of tautomycetin BGC was proved to stably maintain two BGCs in the presence of appropriate antibiotic selection (Nah et al., 2015).

Meanwhile, the TAR system used yeast homologous recombination-based promoter engineering for the activation of silent natural product BGCs (Montiel et al., 2015). Bi-directional promoter cassettes were generated by PCR amplification of varied yeast selectable markers, which contains promoter-insulator-RBS combinations, and they were co-transformed with the cosmid or BAC clone harboring the target BGC into yeast. The rebeccamycin BGC was used as a model BGC. The promoter-replaced rebeccamycin BGC was transferred into *S. albus* by conjugation, and the production of rebeccamycin was examined in the heterologous host (Montiel et al., 2015). Using the TAR-based promoter engineering strategy, multiple promoter cassettes could be inserted simultaneously into the target BGC, thereby expediting the re-engineering process. The TAR-based promoter engineering strategy was also used to capture the silent tetarimycin BGC and the silent, cryptic pseudogene-containing, environmental DNA-derived lazarimide BGC (Montiel et al., 2015).

In conclusion, *Streptomyces* heterologous expression systems have been proved to be a very attractive strategy to awaken cryptic NP BGCs, and could also be applied to overexpression of a variety of large NP BGCs in actinomycetes.

## Author contributions

HN, SK, SC, and EK planned, outlined, and revised the manuscript. HN, HP, and EK wrote and revised the manuscript.

### Conflict of interest statement

The authors declare that the research was conducted in the absence of any commercial or financial relationships that could be construed as a potential conflict of interest.
